# Unveiling the Layers of Borderline Personality Disorder: A Systematic Review of Clinical Subtypes

**DOI:** 10.3390/bs15070928

**Published:** 2025-07-09

**Authors:** Alexandra Triantafyllou, Pentagiotissa Stefanatou, George Konstantakopoulos, Eleni Giannoulis, Ioannis Malogiannis

**Affiliations:** 1 First Department of Psychiatry, Medical School, National and Kapodistrian University of Athens, 11528 Athens, Greece; 2 Department of Speech and Language Therapy, University of Peloponnese, 24150 Kalamata, Greece; 3 Research Department of Clinical, Education and Health Psychology, University College London, London WC1E 7HB, UK

**Keywords:** borderline personality disorder, cluster analysis, subgroups, subtypes, systematic review

## Abstract

**Background:** Borderline personality disorder (BPD) is characterised by significant clinical heterogeneity. Classifying subtypes of BPD may offer deeper insights into the disorder’s complexity and inform more tailored therapeutic strategies. The exploration of data-driven subtyping using cluster-analytic approaches represents a promising avenue for capturing variability in symptomatology and comorbidity profiles. **Aim:** This systematic review aims to synthesise and critically evaluate the empirical studies that have applied cluster-analytic methods to identify subtypes of BPD in adult populations. It further assesses the consistency of findings and their alignment with theoretical models of the disorder. **Methods:** A comprehensive search of PubMed, Scopus, and PsycNet was conducted in accordance with the PRISMA guidelines. Eligible studies employed either traditional or probabilistic clustering techniques to classify adult individuals diagnosed with BPD based on the DSM criteria. A total of 29 studies, encompassing 24,345 participants, met the inclusion criteria. The study quality and risk of bias were assessed using the AXIS tool. **Results:** Most studies identified clinically meaningful BPD subtypes based on dimensions such as affective regulation, effortful control, interpersonal style, and impulsivity or aggression. Several findings supported the existence of internalizing and externalizing profiles, which converge with long-standing theoretical conceptualisations of BPD. However, substantial heterogeneity was observed in subtyping bases, sample characteristics, and analytic procedures. **Discussion:** Although this review highlights the recurring subtype patterns, the methodological inconsistencies and a lack of longitudinal and treatment-outcome data limit the strength of the conclusions. The future research should prioritise standardised subtyping frameworks and explore the prognostic and therapeutic utility of BPD subtypes in clinical settings.

## 1. Introduction

### 1.1. Borderline Heterogeneity: Two Approaches

According to the DSM-5 (APA, 2013), borderline personality disorder is defined as a pervasive pattern of instability in self-image, affect, and interpersonal relationships, accompanied by impulsivity and self-destructive behaviours. The diagnosis was introduced in DSM-III (APA, 1980) and since then, many studies have focused on exploring the heterogeneity of the disorder through its dimensions and subtypes within the DSM criteria. The DSM-5 diagnostic criteria (APA, 2013 for BPD present BPD as a single, unified diagnostic entity without any mention of clinical subtypes, as is found in other mental disorders. Although the DSM-5 acknowledges that patients with BPD may present with varying combinations of the nine diagnostic criteria, leading to a heterogeneous clinical picture, it does not systematize these variations into subtypes. This might mean that the existing research findings on the subtypes were not robust enough, or present significant contradictions, and therefore, no subtyping could be accepted.

The research on the heterogeneity of borderline personality disorder (BPD) has taken two main approaches: examining the dimensions underlying the BPD criteria using factor analysis and identifying potential subtypes using cluster analysis. The first is variable-oriented, focusing on the DSM criteria, while the second is person-centred (Hallquist & Pilkonis, 2012).

Exploratory methods used by person-centred studies exploring BPD heterogeneity include traditional methods and probabilistic methods. Cluster analysis methods are exploratory techniques designed to identify groups in data based on proximity measures, as opposed to discriminatory methods with pre-defined groups. The two main traditional methods are hierarchical cluster analysis, which creates a hierarchy without fixed clusters, and clustering optimization algorithms such as k-means, which assign individuals to a fixed number of groups. Both are heuristic and do not assume any class structure, unlike newer probabilistic models that propose the existence of subpopulations with different multivariate probability densities. Probabilistic methods include latent class analysis (LCA) and latent profile analysis (LPA) (Landau et al., 2011).

The advantages of model selection include objective procedures, individual classification probabilities, and parameters for clustering new individuals (Kent et al., 2014). LCA is for categorical variables, while LPA is for continuous variables. SPSS two-step cluster analysis addresses the limitations of traditional methods by pre-clustering dense regions and using a hierarchical technique that allows statistical testing and confidence calculations (Bacher et al., 2004).

### 1.2. Borderline Heterogeneity Through BPD Dimensions

The research into the heterogeneity of BPD has focused on the dimensions proposed by Linehan (1993) and others (Gunderson, 2010; Lieb et al., 2004). There is consensus on four behavioural domains: impulsivity and self-injury, affective symptoms, identity disturbance (often associated with emptiness or paranoia), and unstable relationships with fears of abandonment. Factor analyses of these dimensions have yielded mixed results, with many studies supporting a unidimensional model (Nestadt et al., 2006; Hallquist & Pilkonis, 2012; Sharp et al., 2015; Mneimne et al., 2021).

Several studies have supported two-dimensional (Rosenberger & Miller, 1989; Whewell et al., 2000) or three-dimensional models (Livesley & Schroeder, 1991; Sanislow et al., 2000). The most prominent is a three-factor model by Sanislow et al. (2000, 2002), which includes impaired relatedness, affective dysregulation, and behavioural dysregulation. Independent research supports this model (Andión et al., 2011; Calvo et al., 2012). Some research has also identified dimensional subtypes in individuals with BPD (Rusch et al., 1992; Johnson & Levy, 2020), linking these factors to specific subgroups. A review of the evidence regarding BPD dimensions suggests that an understanding of BPD as a unified diagnosis composed of three underlying dimensions could help in targeting different symptom areas while maintaining a clinically meaningful diagnosis (Triantafyllou et al., 2025).

Another source of heterogeneity within the criteria is the fact that BPD appears to lie on the border between internalizing and externalizing disorders (Krueger, 1999; Eaton et al., 2011; Kotov et al., 2011), showing associations with both the distress subfactor of the internalizing dimension and the externalizing dimension (Eaton et al., 2011) which might be better understood through the Hierarchical Taxonomy of Psychopathology (HiTOP) model. Unlike the DSM-5, which uses categorical diagnoses, the HiTOP model (Krueger et al., 2021) offers a dimensional, hierarchical approach to understanding mental disorders, focusing on spectrum-level traits and symptom clusters that cut across traditional diagnoses. Therefore, HiTOP does not treat BPD as a discrete disorder but as a configuration of maladaptive traits and symptom dimensions and as BPD is primarily situated within the “Internalizing” and “Externalizing” spectra, researchers have suggested different BPD components based on HiTOP’s dimensions: an internalizing-dominant component of BPD exhibiting high emotional distress, mood instability, depression, anxiety, and self-harm without significant externalizing behaviours, an externalizing-dominant component of BPD exhibiting impulsivity, anger, substance use, and interpersonal aggression, and a mixed component of BPD exhibiting significant features from both the internalizing and externalizing spectra (Krueger et al., 2021).

### 1.3. BPD Theoretical Subtypes

BPD theorists have identified several subtypes of the disorder regarding adults. Blatt and Auerbach (1988) distinguished two stable types (anaclitic and introjective) and a schizophrenia-like subtype.

Linehan (1993) categorized them according to treatment behaviour: “Butterfly” patients struggle to engage in therapy, whereas “attached” patients bond quickly with therapists and attend sessions consistently. Cohen and Sherwood (1996) identified a quiet borderline patient who is compliant and insecure, and a dramatic subtype who is outwardly demanding. Layden et al. (1993) focused on traits from other personality disorders to define avoidant/dependent, histrionic/narcissistic, and antisocial/paranoid subtypes. Millon et al. (2012) proposed four additional subtypes based on similar traits: discouraged (avoidant/depressive), impulsive (histrionic/antisocial), petulant (passive-aggressive), and self-destructive (depressive/masochistic). Oldham identified five BPD subtypes—affective, impulsive, aggressive, dependent, and empty—based on their aetiology. He recommended that psychotherapy and pharmacotherapy be tailored to better address the symptoms of each subtype (Oldham, 2006).

Although several theoretical subtypes of BPD—such as internalizing vs. externalizing or affective vs. impulsive—have been proposed over the past several decades, their empirical validation remains inconsistent and fragmented. The existence of Blatt and Auerbach’s (1988) subtypes have been supported in a study by Blatt et al. (1988) in a mixed sample of psychiatric patients with severe personality disorders and schizophrenia. Linehan’s subtypes have been supported by Leihener et al. (2003) and Ryan and Shean (2007), while Oldham’s subtypes have been partially supported by a study using participant assignment to groups (Rebok et al., 2015).

This review addresses a critical gap in the literature by systematically synthesising the cluster-analytic studies that have attempted to derive BPD subtypes empirically in adult populations. This review’s novelty lies in its comparative approach: rather than presenting a descriptive list of subtypes, it evaluates the extent to which empirical findings support, refine, or challenge the long-standing theoretical models.

Importantly, comparing data-driven and theory-driven classifications provides a deeper understanding of the conceptual validity and clinical applicability of subtype frameworks. By identifying areas of agreement and disagreement between theory and empirical findings, this review helps clarifies which subtypes are most supported, where inconsistencies lie, and how future studies might reconcile these differences. In doing so, this review systematises the existing evidence into a dimensional structure (e.g., internalizing–externalizing, severity, comorbidity), critically appraises the methodological and conceptual foundations of each subtype model, and highlights the implications for diagnosis, prognosis, and treatment planning. To our knowledge, no previous review has undertaken this comparative and integrative task using a structured quality appraisal (AXIS) and a dimension-based synthesis across 29 studies.

### 1.4. Subtypes Across Development and Clinical Characteristics

#### 1.4.1. Subtypes in Adolescents

The research on BPD in adolescents primarily follows the internalizing–externalizing typology. Early work by Goodrich and Fullerton (1984) identified antisocial and schizoid/withdrawn profiles. Bradley et al. (2005) then expanded this model to include high-functioning internalizing, histrionic, depressive/internalizing, and angry/externalizing subtypes. These categories were later adapted for adult populations (Conklin et al., 2006), reflecting continuities in symptom expression throughout development. More recent studies have provided empirical support for internalizing and externalizing subtypes in adolescents (Ramos et al., 2014; Kalpakci et al., 2018). Using ICD-10-based diagnosis, Cavelti et al. (2021) confirmed the clinical relevance of such symptom profiles in young people.

#### 1.4.2. Subtypes in Adults Based on Comorbidity

In adult populations, subtypes have been proposed based on psychiatric and medical comorbidities. For example, Ferrer et al. (2010) identified a subtype characterised by impulsivity and ADHD comorbidity, while Prada et al. (2014) found elevated substance use and reduced inhibition in individuals with a BPD–ADHD overlap. Howard et al. (2021) described an antisocial subtype characterised by increased substance misuse and legal problems. Furthermore, the presence of organic or neurological comorbidities has been employed to distinguish BPD subgroups, as demonstrated in earlier studies by Andrulonis et al. (1982) and Van Reekum et al. (1993).

#### 1.4.3. Subtypes in Adults Based on Severity

Another stream of research has differentiated BPD subtypes based on severity. Zanarini (2005) conceptualised this dimensionally, and empirical studies have confirmed that individuals who meet more DSM-5 criteria exhibit more severe functional impairment (e.g., Marziali et al., 1994; Asnaani et al., 2007; Dévieux et al., 2009). Similarly, cluster analysis studies have similarly found support for severity-based distinctions in both clinical and subclinical populations (Shevlin et al., 2007; Clifton & Pilkonis, 2007; Thatcher et al., 2005; Fossati et al., 1999; Bornovalova et al., 2010). These findings are consistent with dimensional models such as the HiTOP framework, emphasising the importance of severity in the conceptualisation and planning of interventions for BPD.

### 1.5. Aim and Scope

Given the marked heterogeneity of BPD, several studies throughout its formation as a psychiatric diagnosis have aimed to address the possible existence of subtypes, the vast majority of which have used cluster-analytic methods. Though the research conducted in this area could prove to be beneficial in better understanding and treating BPD, different study designs, participants’ characteristics, and subtyping bases employed, in addition to the lack of statistical power of some of the studies, make it difficult to extract meaningful conclusions regarding the existence and treatment of BPD subtypes. While both the variable-centred and the person-centred approach are valuable in understanding borderline heterogeneity, the present review focuses on the analysis of studies using a person-centred approach, linking their findings to proposed dimensions.

Cluster analysis is a group of statistical techniques that categorise individuals based on shared characteristics, without relying on predefined diagnostic categories. These person-centred methods, whether heuristic (e.g., k-means clustering or hierarchical clustering), or probabilistic (e.g., latent class analysis (LCA), or latent profile analysis (LPA)), are particularly suited for identifying subtypes within heterogeneous populations such as those with borderline personality disorder (BPD). Unlike variable-centred approaches which test associations between symptoms, cluster analysis allows for the empirical identification of naturally occurring patient subgroups, each with a distinct clinical, emotional, and functional profile. This has direct implications for refining diagnostic formulations and developing more personalised treatment pathways.

This systematic review contributes to the field in two key ways. Firstly, it is the first to synthesise the cluster-analytic studies on BPD subtypes in a manner that critically compares their results with long-standing theoretical models. By mapping empirical clusters onto conceptual frameworks (e.g., internalizing vs. externalizing, impulsive vs. affective), it evaluates whether proposed typologies are supported by the data. Secondly, this review offers a structured synthesis that highlights patterns of convergence across diverse methods and populations. This advances both theoretical understanding and informs clinical decision-making by identifying subtype-specific symptom patterns and comorbidities that can inform intervention planning. By doing so, this review paves the way for a more nuanced, evidence-based approach to personalised care in BPD.

## 2. Methods

This systematic review follows PRISMA guidelines (Page et al., 2021) and did not require ethical approval. Risk of bias for each included study was assessed by two reviewers using the Appraisal Tool for cross-sectional studies (AXIS) (Downes et al., 2016). The present review primarily seeks to explore whether BPD subtyping research has reached a relative consensus regarding the existence, number, and form of borderline subtypes. Furthermore, this study explores which subtyping bases are used with more frequency to classify individuals into subtypes. The extracted data will be synthesized narratively based on subtyping findings, aiming at achieving more coherent comparisons between studies, and identifying common themes emerging from groups of studies using different subtyping perspectives. Quantitative methods of interpreting the results were not feasible due to methodological heterogeneity of the studies, including differences in sample characteristics and measurement instruments, which would have limited meaningful integration of results.

### 2.1. Eligibility Criteria

Eligible studies were empirical, quantitative research papers published in English-language, peer-reviewed journals. The primary aim of the studies was to identify and classify subtypes of borderline personality disorder (BPD) in adult populations using cluster-analytic methods. Both traditional clustering techniques (e.g., k-means clustering and hierarchical clustering) and probabilistic/model-based methods (e.g., latent class analysis [LCA], latent profile analysis [LPA], and finite mixture modelling [FMM]) were accepted, provided they were used to derive subtypes based on the criteria for BPD defined in the DSM.

To ensure methodological comparability, studies were only included only if they met the following criteria:

- used a person-centred, data-driven cluster analysis approach (traditional or probabilistic);
- examined subtypes of BPD defined according to DSM diagnostic criteria;
- included adult samples (mean age ≥18 years);
- enrolled a minimum of 40 participants.

Studies were excluded if they:

- relied solely on clinical judgment or variable-centred statistical techniques (e.g., factor analysis);
- focused on non-DSM constructs or non-BPD populations (e.g., adolescents, trait-based samples, general personality pathology);
- had sample sizes smaller than 40 participants;
- were review articles, qualitative studies, opinion pieces, or case reports.

While some frequency analysis studies and theoretical papers are referenced in the discussion for context, they were not included in the formal synthesis.

### 2.2. Search Strategy

A total of 3760 articles were identified and stored in the reference management program Zotero in the search conducted in June 2023 and concluded on 10 June 2023. There was no time limit set on searches in order to capture early date data regarding BPD subtypes. Three computerized databases were searched (PubMed, Scopus, PsycNet). A further two articles were identified by searching article citations.

The search terms were: (“borderline personality disorder”) AND (subtyp* OR subgroup* OR subcategor* OR subpopulation* OR “latent class*” OR “cluster analysis*” OR profil* OR phenotype* OR heterogene*).

Inclusion at the title/abstract stage was conservative (leaning towards inclusion) to ensure the inclusion of all relevant articles. One reviewer screened excluded articles, and three reviewers working in collaboration confirmed that the included articles met the inclusion and exclusion criteria and ensured that all relevant data collected from the articles were accurate.

For each included article, data were collected regarding participant characteristics, subtyping basis and tools used, and number and characteristics of subtypes.

### 2.3. Study Quality Assessment

The AXIS tool was used to assess the quality of each study across 20 criteria, including sample selection, clarity of reporting, and methodological rigour. Each study was rated independently by two reviewers, and any discrepancies were resolved through discussion. Although studies were not excluded based on their AXIS score, the quality assessments informed the synthesis and interpretation of the results. Specifically, studies that met at least 15 criteria were considered to be of a higher quality and were given greater weight when identifying consistent subtype patterns. Findings from studies meeting fewer than 10 AXIS criteria were treated with caution, and any conclusions drawn from such studies were presented as preliminary or hypothesis-generating, unless corroborated by higher-quality research.

### 2.4. Classification and Interpretation of Clustering Methods

In this review, we included both traditional clustering methods, such as hierarchical clustering, k-means clustering, and two-step clustering), and probabilistic/model-based clustering methods, such as latent class analysis (LCA), latent profile analysis (LPA), and finite mixture modelling (FMM), because they all aim to identify subgroups within heterogeneous populations. Despite being grouped under the umbrella of “cluster-analytic” methods, these approaches differ significantly in their conceptual underpinnings and statistical assumptions.

Traditional clustering methods are heuristic and use proximity-based measures (e.g., Euclidean distance) to partition individuals into subgroups, making no assumptions about an underlying distribution. These methods are flexible, but they often depend on subjective decisions, such as the number of clusters or the linkage method. In contrast, probabilistic or model-based methods are grounded in statistical theory and assume that the observed data arise from distinct latent classes, each with a specific probability distribution. These approaches offer formal model-fit indices (e.g., AIC, BIC, and entropy) and allow for probabilistic classification, making them more robust in terms of reproducibility and interpretive clarity.

We chose to include both types of methods in order to capture the full range of empirical efforts to subtype BPD. However, during synthesis and interpretation, we accounted for methodological heterogeneity. When interpreting convergent patterns, results from probabilistic approaches were given greater weight, especially when based on larger samples and sound model-fit criteria. Conversely, findings from traditional clustering were examined in light of their exploratory flexibility and potential subjectivity. These distinctions are elaborated on further in the Discussion section (Section 4.3), particularly when evaluating the robustness and comparability of the subtype structures identified across studies.

## 3. Results

### 3.1. Study Selection

The database search yielded 3760 reports, which were reduced to 1914 after removing duplicates. Title screening excluded 913 irrelevant articles. In the next stage, 1001 abstracts were screened; 601 were irrelevant and 200 were non-quantitative research. Of the 198 articles requested for retrieval, 3 were unavailable, 4 were in a non-English language, and 8 were not peer-reviewed.

**Figure 1:**
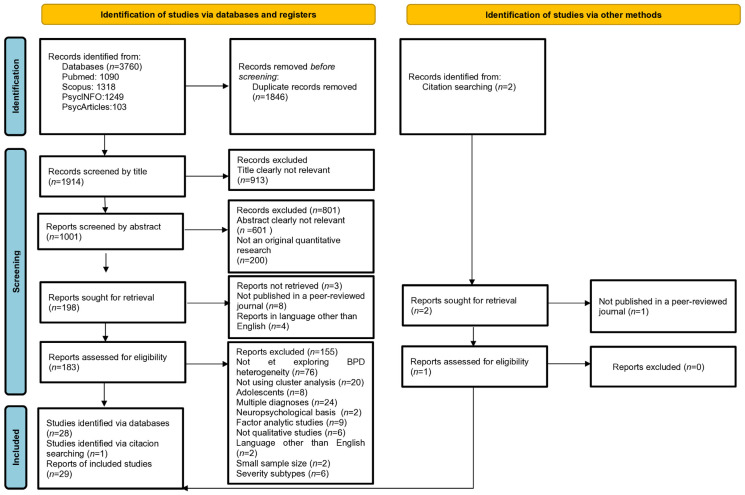
Prisma diagram detailing the study selection process.

A total of 183 articles were assessed, 76 of which did not examine the heterogeneity of BPD. Nine studies focused exclusively on variable-centred perspectives of BPD heterogeneity, while twenty identified subtypes using methods other than cluster analysis. Eight studies included underage samples, six based the subgrouping solely on severity in non-BPD populations, two used general medical or neuropsychological features for subtyping, and twenty-four identified groups with multiple or non-BPD diagnoses. Two studies were excluded due to small sample sizes of less than 40 participants, as a total sample of less than 40 participants may not achieve sufficient statistical power (Dalmaijer et al., 2022). In addition, two non-English studies led to the exclusion of a total of one hundred and fifty-five studies at the eligibility stage. Two additional studies were found through citation searches, one of which was not peer-reviewed. Finally, 29 studies focusing on BPD subtypes identified by cluster analysis were included in this review (Figure 1).

### 3.2. Study Characteristics

A total of 29 trials with 24,345 participants were included, after accounting for overlap in two trials. Of these, 20,010 participants contributed to a university data repository (Johnson & Levy, 2020). The average study size was 158, excluding the Johnson & Levy study. Twenty-five studies reported gender demographics, mostly female (mean = 80.6%, range = 53.4–100%). Twenty-two studies reported mean age, with an average of 29.4 years. Most studies were conducted in the USA (12 studies), followed by Canada (4), Germany and Australia (3 each), Spain and Belgium (2 each), and one each from the Netherlands, Italy, and Sweden. Most of the samples included people who met BPD criteria; seven did not exclusively include BPD patients.

The studies included different samples: one with inpatients (Barrash et al., 1983), two with moderate BPD pathology (Wright et al., 2013; Gamache et al., 2021), one from the general population (Johnson & Levy, 2020), one primarily with individuals with personality disorders meeting the BPD criteria (Jaeger et al., 2017), and one from a BPD treatment centre, not all of whom met the full criteria (Andión et al., 2013). One study recruited people with BPD or sub-threshold BPD online (Hanegraaf et al., 2023). Five studies included inpatients, and seven studies focused on specialized PD settings (Table 1).

The study quality was assessed using the Appraisal Tool for Cross-Sectional Studies (AXIS) (Downes et al., 2016) to ensure an appropriate design for reliable results. Most studies were of moderate quality, with common problems relating to non-representative convenience samples from psychiatric settings. Only two studies justified their sample size. Ethical approval and disclosure of conflicts of interest were often lacking, especially in older studies. Overall, only 17.24% of studies met at least 15 AXIS criteria, indicating high quality; most met between 10 and 15 criteria; meanwhile, two met fewer than 10, indicating moderate to low study quality (Table A1). No studies were excluded due to their AXIS score, taking under consideration the decision to include older studies which resulted in studies not meeting AXIS criteria since completion of specific requirements was not common practice in scientific research of past decades (e.g., declaration of competing interests) and also taking under consideration that the reduced statistical power due to small and heterogeneous samples reflects the challenges imposed by the research on clinical populations.

### 3.3. Methods Used in Studies of BPD Subtypes

In this review, five studies used hierarchical clustering to subtype BPD (Leihener et al., 2003; Ryan & Shean, 2007; Salzer et al., 2013; Gamache et al., 2018; Oladottir et al., 2022), three studies determined the number of subgroups as input for k-means cluster analysis (Frias et al., 2017; Smits et al., 2017; Magni et al., 2021), four applied pure k-means (Barrash et al., 1983; Hoermann et al., 2005; Andión et al., 2013; Hanegraaf et al., 2023), and four used SPSS two-step cluster analysis (Digre et al., 2009; Gamache et al., 2018; Sleuwaegen et al., 2017, 2018).

Eleven studies used probabilistic methods with increasing frequency over the last decade: two used finite mixture modelling (Lenzenweger et al., 2008; Hallquist & Pilkonis, 2012), four used latent class analysis (Wright et al., 2013; Johnson & Levy, 2020; Black et al., 2022; Antoine et al., 2023), and another four used latent profile analysis (Newhill et al., 2010; Jaeger et al., 2017; Gamache et al., 2021; Aleva et al., 2023).

Growth mixture modelling (McMain et al., 2018) differs from latent class analysis in that it uses categorical variables, whereas latent profile analysis uses continuous variables. Critchfield et al. (2008) used q-factoring to group participants by profile similarity into a q-matrix for factor analysis. Soloff and Chiappetta (2012) applied trajectory analysis to reveal longitudinal patterns in subgroups, similar to finite mixture methods.

### 3.4. Basis for Subtyping and Number of Subtypes

#### 3.4.1. Subtyping Based on DSM Criteria

Four studies have used DSM criteria to subtype individuals with BPD (Barrash et al., 1983; Andión et al., 2013; Johnson & Levy, 2020; Antoine et al., 2023). Barrash et al. (1983) initially identified two subtypes: borderline type 1, with schizotypal traits, and the rarer type 2, with purely borderline traits. Later research has shifted the focus of subtyping to dimensional aspects of BPD. Andión et al. (2013) examined Sanislow et al.’s (2000, 2002) model, confirming five classes of participants based on the dimensions of the disorder: relatedness, behavioural dysregulation, affective dysregulation, a class with all dimensions present, and a class with none.

Johnson and Levy (2020) identified four BPD classes in a community sample: asymptomatic, affective/impulsive, empty/identity disordered, and a general ‘BPD’ class. Their factor mixture modelling proposed an unstable and empty phenotype with three classes: asymptomatic, empty, and unstable. Similarly, Antoine et al. (2023) found interpersonally unstable and impulsive subtypes in addition to a dissociative subtype. Black et al. (2022) also categorized individuals on the basis of BPD criteria into a blank/dissociative group and an affective instability/substance abuse group.

Other studies using non-cluster analytic methods have used factor analysis to identify different subgroups within BPD. Rusch et al. (1992) identified four subtypes, including unstable and identity types; meanwhile, Whewell et al. (2000) recognised similar categories along with an undifferentiated type and internalizing/externalizing types based on specific criteria met. Finally, Lewis et al. (2012) identified three distinct groups—emotionally dysregulated, rejection sensitive, and mentalisation failure—through their analysis of DSM criteria.

While studies have differed on the number and nature of BPD subtypes, four studies have supported a subtype associated with affective instability. In addition, some studies have supported an identity disorder subset similar to Linehan’s “disorder of the self”. There are also behavioral dysregulation subgroups related to impulsivity and self-injury, although there is disagreement about whether impulsivity correlates with emotional liability in BPD or represents distinct subtypes. Three studies used the DSM-5 alternative model for personality disorders, which identifies four profiles: asymptomatic, moderate pathology with impulsivity, moderate pathology with identity disturbance, and severe pathology.

Based on the alternative model for personality disorders, Gamache et al. (2021) identified four profiles based on self and interpersonal functioning, relating those who exhibit impulsivity as externalizing subtypes and those with identity disturbance as internalizing subtypes, and in the same line of thinking, Hanegraaf et al. (2023) identified two internalizing and one externalizing subtypes of BPD, while Frias et al. (2017) found hostile, self-sufficient, dependent, and suspicious types based on dimensional personality traits.

#### 3.4.2. Subtyping Based on Prominent Borderline Traits

Two studies identified clusters based on features of other personality disorders. Critchfield et al. (2008) found narcissistic/histrionic and avoidant/obsessive–compulsive profiles, as well as a rare paranoid/schizotypal profile. Smits et al. (2017) confirmed the paranoid/schizotypal profile and introduced an extravert/externalizing subgroup but did not find a borderline group with avoidant/obsessive-compulsive traits, instead proposing a ‘core BPD’ group.

While both studies supported a schizotypal/paranoid subtype, Layden et al. (1993) and Millon et al. (2012) did not include schizotypal traits in their theoretical subtypes, while the findings regarding a histrionic/externalizing subtype (Critchfield et al., 2008; Smits et al., 2017) are in line with the theoretical histrionic-narcissistic (Layden et al., 1993) or impulsive (Millon et al., 2012) subtype. Although avoidant/dependent subtypes have been mentioned theoretically, only Critchfield et al.’s research has verified internalizing features in a specific subtype.

Five studies have examined subtypes based on emotional regulation and effortful control. The first study (Hoermann et al., 2005) identified three subgroups. The research has suggested that emotional control in individuals with BPD correlates with lower psychopathology, better adaptive defences, and less social alienation. Rufino et al. (2017) identified three subgroups of BPD based on emotional regulation, linking better regulation to improved functioning. Aleva et al. (2023) also found three subgroups of emotional regulation but noted that emotional awareness does not guarantee effective regulation. Sleuwaegen et al. (2017, 2018) proposed three emotion subtypes—low anxiety, inhibited, and disinhibited—and noted a high self-control subtype that was not confirmed in later research. Digre et al. (2009) categorized people with BPD into groups based on their emotional contribution styles: withdrawn–internalizing, severely disturbed–externalizing, and anxious–externalizing. Collectively, these studies have highlighted the importance of emotional regulation for the functioning and psychological well-being of people with BPD while emphasizing the role of specific regulatory strategies in differentiating subtypes. These findings also imply that emotional liability, a key feature of the disorder (Dreyße et al., 2020; Peters et al., 2022), is not present in all subtypes.

Four studies identified BPD subtypes based on interpersonal problems, using the Inventory of Interpersonal Problems (IIP). Two studies followed Marsha Linehan’s classification of ‘butterfly’ and ‘attached’ types, identifying an autonomous and a rare dependent subtype (Leihener et al., 2003; Ryan & Shean, 2007). Salzer et al. (2013) identified five classes, while Wright et al. (2013) recognized six.

In addition, three studies emphasized aggressive, antisocial, paranoid, or psychopathic traits in BPD subtyping. Lenzenweger et al. (2008) found three groups based on these traits; Newhill et al. (2010) identified four related to psychopathy; Hallquist and Pilkonis (2012) distinguished four based on anger and trust.

Although differences in measures and designs prevent direct comparisons of findings regarding aggressive traits across BPD subtypes, it’s clear that aggression is related to other personality traits and varies across subtypes. Studies that focus on antagonistic traits tend to identify fewer but more socially adapted subtypes of the disorder.

## 4. Discussion

This review aimed to systematically identify and evaluate empirical studies applying cluster-analytic methods to delineate clinical subtypes of borderline personality disorder (BPD) in adults. The primary objective was fourfold: (1) to assess the heterogeneity of the methodologies used to derive subtypes; (2) to synthesise and compare the resulting subtype structures across studies; (3) to evaluate the extent to which these empirically derived subtypes align with existing theoretical models; and (4) to discuss the clinical implications of these findings for diagnosis and personalised intervention strategies. In this section, we examine whether these aims were achieved and what novel insights have emerged.

### 4.1. Summary of Key Findings

This review systematically examined the application of cluster-analytic methods to identify subtypes of borderline personality disorder (BPD) in adults. Despite methodological diversity, the findings indicate, convergence around certain subtype structures, most notably internalizing/externalizing profiles and severity gradients. These results support the view that BPD is not a monolithic diagnosis, but rather a heterogeneous syndrome with clinically meaningful subgroups.

### 4.2. Understanding BPD Subtyping on the Dimensions of Internalization-Externalization and Severity

The studies in this review used different research designs and focused on different aspects. Personality traits show considerable diversity, but little effort has been made to replicate study findings across samples. Digre et al. (2009), Johnson and Levy (2020), Gamache et al. (2021), Oladottir et al. (2022), and Hanegraaf et al. (2023) categorized their subtypes on the internalization–externalization continuum and noted their names. These studies used different bases for the externalizing and internalizing subtypes. Therefore, it should not be assumed that the subtypes correspond. Most studies show different subtypes with different characteristics.

The research on borderline personality disorder (BPD) suggests distinct internalizing and externalizing profiles. Initial studies by Barrash et al. (1983) highlighted the isolation of schizotypal borderline type 1 from the unstable relationships of type 2. Subsequent findings by Critchfield et al. (2008) and Smits et al. (2017) revealed a paranoid/schizotypal profile associated with social isolation, while Johnson and Levy (2020) found that their empty and unstable groups exhibited internalizing and externalizing traits, respectively. Oladottir et al. (2022) identified an impulsive, aggressive externalizing group among those with BPD, contrasting with a dysphoric, stressed internalizing group. Gamache et al. (2021) linked impulsivity and identity issues to the internalization–externalization dimension, while Hanegraaf et al. (2023) found two internalizing subtypes alongside one externalizing subtype of BPD. Sleuwaegen et al. (2017, 2018) also identified an inhibited subtype related to emotional regulation in their studies of BPD subtypes.

The research has identified BPD subtypes based on emotional regulation, distinguishing between internalizing (more emotional control) and externalizing (emotional expression) strategies. Digre et al. (2009) categorized two internalizing groups and one externalizing group. Leihener et al. (2003) and Ryan and Shean (2007) confirmed Linehan’s (1993) autonomous and dependent subtypes in this continuum, while Salzer et al. (2013) and Wright et al. (2013) described an avoidant class.

This classification is consistent with theoretical frameworks that define calm, discouraged, and attached versus dramatic, impulsive/aggressive BPD types. It is also consistent with the understanding of BPD as consisting of an externalizing and an internalizing component as the HiTOP model (Krueger et al., 2021) suggests. However, the research has highlighted that BPD encompasses both symptom types on a spectrum rather than strict categories. Nonetheless, while viewing BPD subtypes in contrasting terms provides a simple and practical way of understanding BPD heterogeneity, it is important to underscore that as the research suggests (Eaton et al., 2011), the nature of BPD incorporates both internalizing and externalizing symptoms, even though some individuals with the disorder seem to be located close to one end of the spectrum.

Severity is another critical dimension; many studies associated emotional dysregulation with different levels of dysfunction. Less emotionally regulated individuals show more severe psychopathology and dysfunction, as shown by Hoermann et al. (2005), Sleuwaegen et al. (2017), and Rufino et al. (2017).

Digre et al. (2009) identified a severely disturbed group with dysfunctional defences and significant psychopathology. Lenzenweger et al. (2008) found a less severe subtype with antisocial, aggressive, and paranoid traits but better adjustment. Hallquist and Pilkonis (2012) distinguished between an angry, aggressive group with high borderline psychopathology and another with fewer antisocial traits. Studies by Magni et al. (2021) and Black et al. (2022) identified severely distressed subtypes based on general psychopathology, while Andión et al. (2013) and Jaeger et al. (2017) focused on high- and low-symptom classes within borderline symptoms, although none looked exclusively at BPD samples.

Frias et al. (2017) described self-sufficient individuals with lower neuroticism versus a suspicious subtype with poor quality of life based on dimensional traits. Gamache et al. (2021) used the alternative personality disorder model to name severity-based subtypes, while Oladottir et al. (2022) and Hanegraaf et al. (2023) found low levels of psychopathology without identifying a more distressed type.

These findings are consistent with the research showing increased general psychopathology and impaired functioning in BPD subgroups defined by criteria met (Marziali et al., 1994; Asnaani et al., 2007; Dévieux et al., 2009), supporting Zanarini’s (2005) theory of severity subtypes in BPD, and is also in accordance with the understanding of BPD as a dimensional disorder (Krueger et al., 2021).

### 4.3. Targeted Treatment of BPD Subtypes

The efforts to subtype BPD are crucial for tailoring treatments, as most researchers agree that no single treatment is suitable for all patients (Critchfield et al., 2008; Smits et al., 2017). Individualized treatment relies on the identification of BPD subtypes. However, few studies link traits to treatment response (Choi-Kain et al., 2016), with only four studies examining BPD subtype response. Sleuwaegen et al. (2018) found that only the low-anxiety subtype did not significantly benefit from DBT, although dropout rates were similar across subtypes. Digre et al. (2009) found differential responses: the withdrawn/internalizing type showed reduced dissociation, while the anxious/externalizing type improved depressive symptoms; no improvement was seen in the severely disturbed internalizing type. These findings suggest that differential responses necessitate targeted treatments for BPD subgroups. In contrast, Black et al. (2022) reported greater improvement in the high-severity groups after STEPPS than in the low-severity groups.

The study by Darmann et al. (2020), which was not included due to small sample size, found that the high anger, contempt, and disgust group benefitted more from 12 weeks of residential treatment than the low-scoring group, who only improved on the reality test. McMain et al. (2018) identified three BPD subtypes based on response to DBT or GPM therapy, while Gamache et al. (2018) found four classes likely to drop out of treatment, suggesting that baseline characteristics may help clinicians with prognosis.

### 4.4. Methodological Considerations

One challenge in synthesising the literature was the methodological diversity in clustering techniques. While traditional clustering methods offer flexibility, they often lack objective statistical criteria for determining the optimal number of clusters. By contrast, probabilistic methods, by contrast, provide formal model selection metrics and probabilistic class membership assignment, enhancing reproducibility and clarity. These methodological differences influence the interpretability and generalisability of findings. Consequently, we gave more weight to subtype patterns that were replicated across multiple, methodologically rigorous probabilistic studies (e.g., LPA studies with a strong model fit) than to those identified solely through exploratory, traditional clustering. This stratified interpretative approach was essential in mitigating the risks of overgeneralisation and emphasising the most reliable empirical subtype structures.

The heterogeneity in study quality, as assessed by the AXIS tool, directly informed the confidence we placed in the findings. For instance, subtype structures that consistently emerged across high-quality studies rated as such (e.g., Johnson & Levy, 2020; Gamache et al., 2021) were considered as more robust and clinically meaningful. Conversely, subtype configurations reported solely in lower-scoring studies—particularly those with small samples, poor reporting, or unclear analytic methods—were identified explicitly as tentative and not used to support firm conclusions. This weighting approach ensured that our synthesis emphasised reliable patterns without overstating the results of studies with methodological limitations.

### 4.5. Synthesis of Objectives and Implications

This review aimed to explore the empirical literature on borderline personality disorder subtypes derived through cluster-analytic methods, critically evaluate their alignment how these subtypes with existing theoretical models, and assess their implications for clinical classification and treatment. To meet these objectives, this review systematically organised findings from 29 studies involving over 24,000 participants into a structured synthesis based on subtyping dimensions (e.g., internalizing/externalizing, severity, comorbidity). This review demonstrated that while no single typology dominates the field, several empirically supported patterns converge with long-standing theoretical distinctions, particularly in the areas of emotional regulation, interpersonal dysfunction, and impulsivity.

Notably, this review revealed that the internalizing and externalizing profiles appear robust across different clustering approaches and populations, and that severity-based subtyping is both clinically meaningful and consistent with dimensional models such as HiTOP. However, critical gaps remain in the form of a lack of longitudinal data and the limited exploration of treatment response. Unexpectedly, few studies have sought to explicitly test theoretical models, and subtype definitions varied widely in granularity and construct base, highlighting the need for greater standardisation in future BPD subtyping research.

## 5. Conclusions

This review systematically examined the use of cluster-analytic methods to identify subtypes of borderline personality disorder (BPD) in adult populations. In line with its stated objectives, this review (1) evaluated methodological diversity, (2) synthesised and compared subtype structures, (3) assessed their alignment with theoretical models, and (4) highlighted implications for clinical practice. These contributions lay the groundwork for a more nuanced, subtype-informed approach to BPD diagnosis and treatment.

Our findings demonstrate that cluster-analytic approaches provide valuable insights into the heterogeneity of BPD, supporting its conceptualisation as a dimensional and multifaceted condition. This review also shows that the methodological quality and analytic choices significantly affect the structure and interpretability of subtypes. Future research should prioritise longitudinal designs, link subtype classification to treatment outcomes, and improve the reliability and clinical usefulness of the results. Transitioning from categorical diagnosis to personalised, data-driven care represents a promising path forward for both research and clinical application.

## 6. Limitations

The present systematic review included studies with a low AXIS score. While the decision to include these studies was taken in order to ensure that all relevant data would be included, given the challenges imposed by research on clinical populations, the conclusions of this review may have been influenced by research results of low statistical value.

Another limitation of the present review is that only papers written in English were considered for inclusion, although it should be noted that both studies excluded when assessed for eligibility because they were written in a language other than English, would have probably been excluded, as, according to their abstracts, Auffret et al.’s (2017) study examined BPD subtypes in adolescents and Zehl et al.’s (2013) study focused on identifying subtypes in traumatized patients.

## Figures and Tables

**Table 1:** Study characteristics.

Author(s)/ Year/ Country/ Title	Participants and Setting	Subtyping Basis, Method and Data Collection	Principal Findings
Barrash et al. (1983), USA Discriminating borderline disorder from other personality disorders: Cluster analysis of the Diagnostic Interview for Borderlines	A total of 252 hospitalised patients, of whom 18 with BPD (DSM-III) No gender and age information	K-means cluster analysis of DIB answers	A total of 8 clusters of which 2 consisted of BPD subtypes (5 individuals with BPD were not included in either of the clusters) 1. *Borderline type 1-schizotypal* (n = 50% of BPD patients): unsociable, isolated, more psychotic experiences, lower work-related functionality 2. *Borderline type 2- borderline* (n = 22.2% of BPD patients): loneliness intolerance, disturbed relationships, impulsivity Similar percentages of suicidal/self-harm behaviours among groups
Leihener et al. (2003), GermanySubtype differentiation of patients with borderline personality disorder using a circumplex model of interpersonal behavior.	A total of 95 females with BPD (DSM-IV), (mean age = 27.1)	Interpersonal profile. Hierarchical cluster analysis of IIP-D data	Two subgroups: 1. *Autonomous type* (n = 27.3%): cold/distant, non-complaisant, non-submissive, difficulties with intimacy 2. *Dependent subtype* (n = 72.6%): low scores on dominance and arrogance, overly accommodating, submissive, self-sacrificing. Conflict avoidance, overly friendly No significant differences in BPD severity
Hoermann et al. (2005), USA The construct of effortful control: An approach to borderline personality disorder heterogeneity	A total of 47 individuals with BPD (DSM-IV) (87.2% female, mean age = 28.9)	Effortful control. k-means cluster analysis of ATQ data	Three subgroups: 1. The 1st cluster (n = 36.2%): high effortful control, low anxiety, low psychoticism and isolation, low identity disturbance, low usage of immature defences 2. The 2nd cluster (n = 23.4%): high activation control, low inhibitory and attentional control, low anxiety, medium psychoticism, low alienation, medium identity disturbance 3. The 3rd cluster (n = 40.4%): low effortful control, high anxiety, high psychoticism, high alienation, high identity diffusion and primitive defences
Ryan and Shean (2007), USA Patterns of interpersonal behaviours and borderline personality characteristics	A total of 49 students with BPD (DSM-IV) No gender or age information	Interpersonal problems. Hierarchical cluster analysis of IIP data	Leihener et al. (2003) findings confirmed by Ryan and Shean (2007) 1. *Autonomous subtype* (n = 24.5%): domineering, controlling, overly assertive, vindictive, distant, self-centred 2. *Dependent subtype* (n = 75.5%): overly accommodating, submissive, difficulties conveying needs, low confidence It is noted that no clusters were formed in the control group
Critchfield et al. (2008), USA Organization of co-occurring Axis II features in borderline personality disorder	A total of 90 outpatients with BPD (DSM-IV) (92.2% female, mean age = 30.9)	Comorbidity with other PDs. 1. Q-matrix factor analysis with PCA using IPDE data on PD criteria.	1. Three factors accounting for 75% of the variance. 27% of patients unclassified: a. *Narcissistic/histrionic profile* (n = 31.1%) b. *Avoidant/obsessive compulsive profile* (n = 25.6%) c. *Paranoid/schizotypal profile* (n = 11.1%)
Lenzenweger et al. (2008), USA Refining the borderline personality disorder phenotype through finite mixture modelling: Implications for classification	A total of 90 outpatients with BPD (DSM-IV) (92.2% female, mean age = 30.9)	Paranoid, antisocial, and aggressive features. Finite mixture modelling with normal components to the data of IPDE, IPO, and SCID	Three components model: 1. *nonaggressive/nonparanoid/non-antisocial* (40%): higher functionality, lower negative emotionality 2. *paranoid/nonaggressive/non-antisocial* (27.8%): lower social closeness, more experiences of sexual abuse 3. *aggressive/antisocial/nonparanoid* (32.2%): lower self-constraint, more impulsivity, severe identity diffusion, more psychopathic characteristics
Digre et al. (2009), Australia Treatment response in subtypes of borderline personality disorder	A total of 77 individuals (96% with DSM-IV BPD, 96.1% female, mean age = 34) treated in a specialized centre	Contribution style. Two-step cluster analysis of the data of PHI, SCID I, SCID-II BDI-II, DES, WCCL, IPSAQ.	Three clusters including 76 out of 77 participants. 1. *withdrawn–internalizing* (n = 29.9%): internal contributions, fewer Axis I disorders, did not seek social support 2. *Severely disturbed–internalizing* (n = 24.7%): internal attributions, grave psychological impairment and psychopathology, dysfunctional defences 3. *anxious–externalizing* (n = 44.2%): External attributions, seeking social support, more anxiety disorders Treatment response: reduction in dissociative experiences in withdrawn–internalizing type. Reduction of depressive symptoms in anxious–externalizing type. Severely disturbed–internalizing not improved by any measurement.
Newhill et al. (2010), USA Psychopathy scores reveal heterogeneity among patients with borderline personality disorder	A total of 221 hospitalized patients with BPD (DSM-III-R) (53.4% female, mean age = 29.24)	Psychopathic characteristics. Latent profile analysis of PCL-SV data	A 4-class solution (entropy = 0.80). 1. *Impulsive/antisocial* (38%) 2. *Low psychopathy* (24%) 3. *Manipulative/narcissistic* (20%) 4. *High psychopathy/antisociality* (18%)
Hallquist and Pilkonis (2012), USA Refining the phenotype of borderline personality disorder: Diagnostic criteria and beyond	A total of 100 individuals with BPD (DSM-IV), (85% female, mean age = 27.38)	Abandonment avoidance, identity disturbance, inappropriate anger, mistrust, aggressiveness, antisocial and paranoid tendencies. Finite mixture modelling of SCID-II PDs data and IIP.	BLRT favoured a 4-class solution (entropy = 0.83) 1. *angry/mistrustful* (n = 28%): high interpersonal sensitivity 2. *low identity/low anger* (n = 27%): more self-mutilating behaviours 3. *prototypical* (n = 23%): low levels of mistrustfulness, aggressiveness, antisocial behaviour, identity disturbance, abandonment avoidance, moderate levels of anger 4. *angry/aggressive* (n = 20%): high abandonment avoidance, interpersonal ambivalence, antisocial behaviour, disinhibition
Soloff and Chiappetta (2012) USA Subtyping borderline personality disorder by suicidal behaviour	A total of 137 repeat suicide attempters with BPD No gender or mean age information	Suicidal behaviour. Trajectory analysis on data of LRS	Two-group solution. 1. *Low lethality group* (n = 51.1%): more negativism, more substance-use disorders, more antisocial and/or histrionic comorbidity 2. *High Lethality group* (n = 48.9%): more severe BPD, older age, poorer psychosocial functioning
Andión et al. (2013), Spain Exploring the clinical validity of borderline personality disorder components	A total of 365 individuals referred to a specialized in BPD treatment centre (63.8% with DSM-IV BPD, 74% female, mean age = 27.4)	DSM-IV criteria. Κ-means cluster analysis to assign participants to criteria factors previously detected (Andión et al., 2011) (disturbed relatedness, affective dysregulation, behavioural dysregulation)	Five clusters: 1. *All low* (n = 17.3%) 2. *All high* (n = 40.9%) 3. *Disturbed relationships cluster* (n = 14.8%): more avoidant and obsessive-compulsive personality features, more Axis I disorders. Meeting identity disturbance, emptiness, unstable relationships, stress-related paranoia criteria 4. Affective dysregulation cluster (n = 15.1%): more Cluster B PDs. Meeting anger, affective instability, abandonment avoidance criteria 5. Behavioural dysregulation cluster (n = 12.1%): more Cluster B disorders, less affective disorders, more substance use disorders. Meeting impulsivity, suicidal/self-harm criteria
Salzer et al. (2013), Germany Patterns of interpersonal problems in borderline personality disorder	A total of 228 hospitalized patients with BPD (92.6% female, mean age = 31.3)	Interpersonal profile. Hierarchical cluster analysis using two IIP circumplex axes (dominance and nurturance).	A 5-cluster solution selected using the Ward method. 1. *Vindictive* (n = 18.9%) 2. *Moderate submissive* (n = 32.5%) 3. *Non-assertive* (n = 26.3%): higher interpersonal dysphoria, higher symptom index 4. *Exploitable* (n = 13.6%) 5. *Socially Avoidant* (n = 8.8%): higher symptomatology
Wright et al. (2013), USA Clarifying Interpersonal Heterogeneity in Borderline Personality Disorder Using Latent Mixture Modeling	Mixed clinical and community sample of 255 meeting 3 or more DSM-III-R BPD criteria (91% psychiatric patients, 75% female) No mean age information	Interpersonal problems. Structural summary method followed by latent class analysis based on IIP-C octant scales	A 6-class solution was selected (entropy = 0.86) 1. *Non-assertive* (8.6%) 2. *Vindictive* (22.3%) 3. *Avoidant* (19.6%) 4. *Highly exploitable* (6.7%) 5. *Intrusive* (8.6%) 6. *Exploitable* (31.3%)
Frias et al. (2017), Spain Defining subtypes of borderline personality disorder based on underlying dimensional personality traits	A total of 100 diagnosed with BPD (DSM-V) in community mental health centre No gender or mean age information retrieved	Dimensional personality traits. Hierarchical cluster analysis followed by k-means analysis based on NEO-PI-R	A 4-cluster solution supported. 1. *Hostile BPD* (12%): average–high scores in all scales with the exception of agreeableness 2. *Self-sufficient BPD* (34%): lower neuroticism, individualism 3. *Dependent BPD* (25%): average neuroticism, openness to experience and agreeableness, average to high scores in consciousness and extraversion, higher suicidal ideation, more substance abuse 4. *Suspicious BPD* (29%): average–high scores in most scales, with the exception of agreeableness and lower quality of life
Jaeger et al. (2017), Germany Dissociation in patients with borderline personality disorder in acute inpatient care—a latent profile analysis	A total of 103 inpatients with personality disorders (81% with emotionally unstable disorder, 82.5% female, mean age 28.6)	Borderline characteristics and dissociation. Latent profile analysis using BSL-95 and FDS	A 3-class solution best fitted the data. 1. Class 1 (n = 17.5%): high symptomatology, pronounced absorption and derealization/depersonalization, all female 2. Class 2 (51.5%): severe borderline symptoms, less frequent dissociative symptoms than class 1 3. Class 3 (n = 31%): lower psychopathology
Rufino et al. (2017), USA Variations of emotion dysregulation in borderline personality disorder: a latent profile analysis approach with adult psychiatric inpatients	A total of 156 hospitalized patients with BPD (DSM-IV) (61.8% female, mean age = 29.4)	Emotion dysregulation. Latent profile analysis based on DERS	A 3-class solution best fitted the sample. 1. *Emotionally aware group* (29.5%): emotional awareness did not lead to emotional regulation during periods of anxiety 2. *Global dysfunction group* (44.2%): more severe suicidal ideation, low acceptance of emotions, significant interference of emotions with functionality, emotion related impulsivity 3. *Low impairment group* (26.3%): lack of interference of emotions with functioning and goal achievement
Sleuwaegen et al. (2017), Belgium Subtypes in borderline patients based on reactive and regulative temperament	A total of 150 hospitalized in specialized DBT clinic with BPD (DSM-IV) (85.6% female, mean age = 29.3)	Reactive and regulative temperament. Two step cluster analysis based on BIS/BAS and Effortful Control scale of ATQ.	A 4-cluster solution accounting for 53%, 68%, and 59% of the variance in BIS, BAS, and EC scores, respectively. 1. *Low anxiety subtype* (21%): higher comorbidity with AsPD, higher emotional expression and reactivity 2. *Inhibited subtype* (24%): more Cluster C features, less hostility, more internalisation 3. *High self-control subtype* (10%): adaptive defences, less psychopathology 4. *Emotional*/*disinhibited subtype* (45%): more Cluster B features, interpersonal sensitivity, high anxiety
Smits et al. (2017), Netherlands Subtypes of borderline personality disorder patients: a cluster-analytic approach	A total of 187 individuals with BPD (DSM-IV) referred to MBT treatment to specialized centre (88% female, mean age = 29.1)	Other PD features. Hierarchical cluster analysis followed by k-means cluster analysis based on SCID-II	Three clusters accounting for 97.3% of the variance. 1. *Core BPD* (76%): only BPD features, higher psychopathology 2. *Extravert/externalizing* (14%): more antisocial, histrionic, narcissistic features, less Axis I psychopathology, more men than in core BPD 3. *Schizotypal/paranoid* (8%)
Gamache et al. (2018), Canada Premature termination of psychotherapy in patients with borderline personality disorder: a cluster-analytic study.	A total of 56 files of patients with BPD (DIB-R) that dropped-out of therapy (60.7% females, mean age = 35.79)	Treatment prognosis. Hierarchical cluster analysis and two-step cluster analysis based on TARS-PD data and GAF.	A 4-class solution retained. 1. Higher functioning (n = 39.3%): better treatment prognosis 2. Narcissistic features/entitlement (n = 39.3%): low occupation rate 3. Pseudo-normality (n = 12.5%): comorbid antisocial PD with high level of functioning, high employment 4. Highly dysfunctional (n = 8.9%): suspiciousness and social isolation
McMain et al. (2018), Canada Outcome Trajectories and Prognostic Factors for Suicide and Self-Harm Behaviors in Patients With Borderline Personality Disorder Following One Year of Outpatient Psychotherapy	A total of 180 individuals with BPD (female = 86.1%, mean age = 30.4)	Treatment (DBT or GPM) outcome. Growth mixture modelling using SASII data.	A model with three classes best fitted the data. 1. Rapid and recovered (n = 84.7%): lowest suicide/self-harm behaviours at baseline, best outcomes 2. Slow and recovered (n = 8.6%): high rate of suicide/self-harm behaviours at baseline 3. Recovered and relapsed (n = 6.8%): highest rate of suicide/self-harm behaviours at baseline, higher depression, emergency department visits, unemployment
Sleuwaegen et al. (2018), Belgium Do treatment outcomes differ after 3 months DBT inpatient treatment based on borderline personality disorder subtypes?	A total of 145 hospitalized in DBT treatment centre with BPD diagnosis (DSM-IV) (88.3% female, mean age at baseline = 29.7)	Reactive and regulative temperament-duplication of Sleuwaegen et al. (2017). Two step cluster analysis based on BIS/BAS and Effortful Control scale of ATQ.	Better fit for the 3-cluster solution. 1. *Emotional/disinhibited* (15%): high behavioural activation, low effortful control 2. *Low anxiety subtype* (41%): low behavioural inhibition, low behavioural activation, neutral effortful control 3. *Inhibited subtype* (44%): high behavioural inhibition, average effortful control Treatment outcomes: No significant differences at drop-out rates. Low anxiety group showed no significant improvement.
Johnson and Levy (2020), USA Identifying unstable and empty phenotypes of borderline personality through factor mixture modeling in a large nonclinical sample	A sample of 20,010 undergraduates (63.86% female, mean age = 18.8)	DSM-V BPD criteria. 1. Latent class analysis based on MSI-BPD 2. Factor mixture modelling based on MSI-BPD	1. A 4-class solution. a. 1st class: asymptomatic (65.2%): b. 2nd class (19.6%): emotional/impulsive c. 3rd class (7.1%): identity disturbed/chronic feelings of emptiness/dissociative d. 4th class (8.1%): “BPD class”, all symptoms except unstable relationships and self-mutilating behaviours had a possibility of appearance > 0.70 2. 1-factor, 3-classes model. a. Asymptomatic (70%) b. *Unstable group* (18.8%): BPD features excluding emptiness and identity disturbance. More frequent self-mutilation, more anger, impulsivity, and maladaptive defences c. *Emptiness Group* (11.3%): BPD features including emptiness and identity disturbance. More negative emotionality and avoidant attachment Noted that Unstable group exhibited more externalizing symptoms and Empty group exhibited more internalizing symptoms
Magni et al. (2021), Italy Psychopharmacological treatment in borderline personality disorder: A pilot observational study in a real-world setting	A total of 75 inpatients and outpatients with BPD (76% female, mean age = 36.7.	Clinical features. Hierarchical cluster analysis followed by k-means cluster analysis	Two clusters: 1. Cluster 1 (70.7%): higher severity, more inpatients, more psychopathology 2. Cluster 2 (29.3%): lower BPD severity, more outpatients, less psychopathology No significant associations between severity cluster and drugs assumption
Gamache et al. (2021), Canada Latent profiles of patients with borderline pathology based on the alternative DSM-5 model for personality disorders	A total of 221 external patients (60.2% female, mean age = 33.7), from a specialized PD program with at least moderate BPD pathology	Alternative model for PDs (DSM-V). Latent profile analysis based on SIFS and PID-5.	A 4-profile solution: a. Borderline traits (18%, BPD = 0%) b. Moderate pathology with impulsivity (n = 21.3%, 40% with BPD): lower negative emotionality and borderline psychopathology, higher aggressivity c. Moderate pathology with identity problems and depressivity (n = 24.2%, 45.1% with BPD): higher dysphoria and psychopathology d. Severe pathology (n = 36.5%%, 90.1% with BPD) Noted that the impulsivity group has more externalizing features and the identity/depressivity group has more internalizing features
Black et al. (2022) USA Factor structure of borderline personality disorder and response to Systems Training for Emotional Predictability and Problem Solving	A total of 164 participants with BPD (85% female, mean age = 31.0)	Axis I and Axis II psychopathology, BPD criteria. Latent class analysis using SIDP-IV, SCID, ZAN-BPD data.	A 4-class model selected by BIC and AIC. 1. *High severity group* (n = 26%): high comorbidity with Axis II and Axis I disorder, high disturbed relationships, abandonment fears, anger 2. *Low severity group* (n = 30%): lower BPD symptom severity, rates of personality and mood disorders moderately high, low rates of anxiety disorders 3. *Empty/dissociation/identity disturbance group* (n = 27%), relatively high rates of suicide/self-harm, high rates of avoidant and obsessive–compulsive PD features and major depressive disorder, low rates of substance use disorders. 4. *Affective instability/substance abuse group* (n = 16%): anger, emptiness, high rates of bipolar disorder, panic disorder, agoraphobia, bulimia nervosa Treatment outcomes (STEPPS): greater improvement for the high severity group, followed by the affective instability/substance use disorders group and empty/dissociation/identity disturbance group. Low severity group benefitted the least.
Oladottir et al. (2022), Sweden Cluster analysis of personality traits in psychiatric patients with borderline personality disorder	A total of 141 individuals diagnosed with BPD treated in a DBT clinic (88.7% female) No mean age information	Personality traits. Hierarchical cluster analysis based on SSP	Three clusters: 1. *Lower psychopathology cluster* (n = 47.5%): less disturbed emotional regulation, fewer dissociative experiences, lower bitterness, petulance, mistrustfulness, aggression 2. *Externalizing cluster* (n = 19.9%): higher impulsivity, adventure-seeking, aggression 3. *Internalizing cluster* (n = 32.6%): greater psychic anxiety, stress susceptibility, lack of assertiveness, lower adventure seeking
Aleva et al. (2023), Australia Emotion dysregulation in young people with borderline personality disorder: One pattern or distinct subgroups?	A total of 137 individuals diagnosed with BPD (81% female, mean age = 19.1)	Emotion dysregulation. Latent profile analysis based on DERS data.	Three subgroups: 1. Moderate and accepting (n = 43.1%): high emotional acceptance, moderate emotion dysregulation 2. High and aware (n = 40.9%): high emotion dysregulation, high emotional awareness 3. Low and unaware (n = 16.1%): low emotion dysregulation, high emotional unawareness.
Antoine et al. (2023) Canada Subgroups of borderline personality disorder: A latent class analysis	A total of 504 research participants from three studies with BPD (DSM IV), (female 81.3%, mean age = 29.0)	BPD criteria excluding common to all participants criterion 5. Latent class analysis using IPDE data.	A 3-class solution: 1. Non-labile type (n = 10.5%): lack of affective instability and dissociation, impulsiveness 2. Dissociative/paranoid type (n = 55.4%): Low efforts to avoid abandonment, low identity disturbance, emotionally and sexually traumatized 3. Interpersonally unstable type (n = 34.1%): efforts to avoid abandonment, aggressive behaviour
Hanegraaf et al. (2023), Australia Combining novel trait and neurocognitive frameworks to parse heterogeneity in borderline personality disorder	A total of 100 recruited online participants with BPD or sub threshold BPD (79% female) No mean age information	Personality characteristics. K-means cluster analysis using PID-5 data.	A 4-cluster model was chosen, with two internalizing and one externalizing subgroup: 1. Negative affectivity (n = 26%) 2. Detached (n = 35%): impaired psychosocial functioning, higher depression 3. Externalizing (n = 12%): higher antagonism, disinhibition, psychoticism, higher self-harm 4. Low psychopathology (27%)

Index of psychometric measures: Adult Temperament Questionnaire (ATQ: Evans & Rothbart, 2007); Beck Depression Inventory (BDI-II: Beck et al., 1996); Behavioral Inhibition/Behavioral Activation System Scales (BIS/BAS: Carver & White, 1994); Borderline Symptom List short version (BSL-23, Bohus et al., 2007); Diagnostic Interview for Borderlines (DIB: Gunderson et al., 1981); Difficulties in Emotion Regulation Scale (DERS, Gratz & Roemer, 2004); Dissociative Experience Scale (DES: Bernstein & Putnam, 1986); Global Assessment of Functioning (GAF: Spitzer et al., 1996); Fragebogen zu Disoziativen Symtomen (FDS, Freyberger et al., 1998); Internal, Personal and Situational Attribution Questionnaire (IPSAQ: Kinderman & Bentall, 1996); International Personality Disorders Examination (IPDE: Loranger, 1995); Inventory of Interpersonal Problems (IIP: Horowitz et al., 1988); Inventory of Interpersonal Problems-Circumplex (IIP-C, Alden et al., 1990); Inventory of Interpersonal Problems short version (IIP-D: Alden et al., 1990); Inventory of Personality Organization (IPO, Clarkin et al., 2001); Lethality Rating Scale (LRS: Oquendo et al., 2003); McLean Screening Instrument for BPD (MSI-BPD, Zanarini et al., 2003); NEO Personality Inventory Revised (NEO PI-R, Costa & McCrae, 1985); Parasuicide History Interview (PHI: Linehan et al., 1989); Personality Inventory for DSM-5 Faceted Brief Form (PID-5, Maples et al., 2015); Psychopathy Checklist-Screening Version (PCL-SV: Hart et al., 1995); Self and Interpersonal Functioning Scale (SIFS: Gamache et al., 2019); Structured Clinical Interview for DSM-IV Axis I (SCID-I: First et al., 1997a); Structured Clinical Interview for DSM-IV Axis II disorders: (SCID-II: First et al., 1997b); Structured Interview for DSM-III-R Personality (SIDP-R: Pfohl et al., 1989); Suicide Attempt and Self Injurious Behavior Interview (SASII: Linehan et al., 2006); Swedish universities Scales of Personality (SSP, Gustavsson et al., 2000); Treatment Prognosis Scale for Personality Disorders (TARS-PD: Gamache et al., 2017); Zanarini Rating Scale for BPD (ZAN-BPD; Zanarini, 2003); Ways of Coping Checklist (WCCL: Vitaliano et al., 1985).

## Data Availability

No new data were created or analyzed in this study. Data sharing is not applicable to this article.

## Abbreviations

The following abbreviations are used in this manuscript:

DSM	Diagnostic Statistical Manual of Mental Disorders
BPD	Borderline Personality Disorder
HiTOP	Hierarchical Taxonomy of Psychopathology
ADHD	Attention Deficit/Hyperactivity Disorder
PRISMA	Preferred Reporting Items for Systematic Reviews and Meta-Analyses
LCA	Latent Class Analysis
LPA	Latent Profile Analysis

## Appendix A

**Table A1:** AXIS tool.

ITEM	(Barrash et al., 1983)	(Leihener et al., 2003)	(Hoermann et al., 2005)	(Ryan & Shean, 2007)	(Critchfield et al., 2008)	(Lenzenweger et al., 2008)	(Digre et al., 2009)	(Newhill et al., 2010)	(Hallquist & Pilkonis, 2012)	(Soloff & Chiappetta, 2012)	(Andión et al., 2013)	(Salzer et al., 2013)	(Wright et al., 2013)	(Frias et al., 2017)	(Jaeger et al., 2017)
Clearly stated objectives	Y	Y	Y	Y	Y	Y	Y	Y	Y	Y	Y	Y	Y	Y	Y
Appropriate study design	Y	Y	Y	Y	Y	Y	Y	Y	Y	Y	Y	Y	Y	Y	Y
Sample size justification	N	N	N	N	N	N	N	N	N	N	N	N	N	N	N
Population clearly defined	N	Y	Y	N	Y	Y	Y	Y	Y	Y	Y	Y	Y	Y	Y
Representative sample	N	Y	Y	N	Y	Y	Y	Y	Y	Y	N	Y	N	Y	N
Proper selection process	N	N	N	N	N	N	N	N	N	N	N	N	N	N	N
Address non-responders	N	N	N	N	N	N	N	Y	N	N	N	N	N	N	N
Appropriate measures	Y	Y	Y	Y	Y	Y	D	Y	Y	Y	Y	Y	Y	Y	Y
Reliable measures	Y	Y	Y	Y	Y	Y	Y	Y	Y	Y	Y	Y	Y	Y	Y
Precision estimates	Y	Y	Y	Y	Y	Y	N	Y	Y	Y	Y	Y	Y	Y	Y
Sufficient methods description	Y	Y	Y	Y	Y	Y	Y	Y	Y	Y	Y	Y	Y	Y	Y
Data adequately described	N	Y	Y	N	Y	Y	Y	Y	Y	Y	Y	Y	N	Y	Y
Non-responders information	N	N	N	N	N	N	N	N	N	Y	N	N	N	N	N
Results internally consistent	D	Y	Y	D	Y	Y	D	Y	Y	Y	Y	Y	Y	Y	Y
Comprehensive description of results	Y	Y	Y	Y	Y	Y	Y	Y	Y	Y	Y	Y	Y	Y	Y
Results justify conclusions	Y	Y	Y	Y	Y	Y	D	Y	Y	Y	Y	Y	Y	Y	Y
Limitations	N	Y	Y	Y	Y	Y	Y	N	Y	Y	Y	Y	Y	Y	Y
Conflict of interest	N	N	N	N	N	N	N	N	N	N	N	Y	N	N	Y
Ethics approval	D	D	D	D	D	D	D	D	D	Y	Y	D	D	D	Y
**ITEM**	**(Rufino et al., 2017)**	**(Sleuwaegen et al., 2017)**	**(Smits et al., 2017)**	**(Gamache et al., 2018)**	**(McMain et al., 2018)**	**(Sleuwaegen et al., 2018)**	**(Johnson & Levy, 2020)**	**(Magni et al., 2021)**	**(Gamache et al., 2021)**	**(Black et al., 2022)**	**(Oladottir et al., 2022)**	**(Aleva et al., 2023)**	**(Antoine et al., 2023)**	**(Hanegraaf et al., 2023)**	
Clearly stated objectives	Y	Y	Y	Y	Y	Y	Y	Y	Y	Y	Y	Y	Y	Y	
Appropriate study design	Y	Y	Y	Y	Y	Y	Y	Y	Y	Y	Y	Y	Y	Y	
Sample size justification	N	N	N	N	N	N	N	Y	N	N	N	N	N	Y	
Population clearly defined	Y	Y	Y	Y	Y	Y	Y	Y	Y	Y	Y	Y	Y	Y	
Representative sample	Y	Y	Y	Y	Y	Y	N	Y	N	Y	Y	Y	Y	N	
Proper selection process	N	N	N	N	N	N	N	N	N	N	N	N	N	N	
Address non-responders	N	N	N	D	N	N	N	N	N	N	N	N	N	N	
Appropriate measures	Y	Y	Y	Y	Y	Y	Y	Y	Y	Y	Y	Y	Y	D	
Reliable measures	Y	Y	Y	Y	Y	Y	Y	Y	Y	Y	Y	Y	Y	Y	
Precision estimates	Y	Y	Y	Y	Y	Y	Y	Y	Y	Y	Y	Y	Y	Y	
Sufficient methods description	Y	Y	Y	Y	Y	Y	Y	Y	Y	Y	Y	Y	Y	Y	
Data adequately described	Y	Y	Y	Y	Y	Y	Y	Y	Y	N	Y	Y	Y	Y	
Non-responders information	N	N	N	N	N	N	N	N	N	N	N	N	N	N	
Results internally consistent	Y	Y	Y	Y	Y	Y	Y	Y	Y	Y	Y	Y	D	Y	
Comprehensive description of results	Y	Y	Y	Y	Y	Y	Y	Y	Y	Y	Y	Y	Y	Y	
Results justify conclusions	Y	Y	Y	Y	Y	Y	Y	Y	Y	Y	Y	Y	Y	Y	
Limitations	Y	Y	Y	Y	Y	Y	Y	Y	Y	Y	Y	Y	Y	Y	
Conflict of interest	N	Y	Y	Y	N	N	N	Y	Y	Y	N	Y	Y	Y	
Ethics approval	Y	Y	Y	D	D	Y	Y	D	Y	Y	D	Y	Y	Y	
